# Adipose stem cells from type 2 diabetic mice exhibit therapeutic potential in wound healing

**DOI:** 10.1186/s13287-020-01817-1

**Published:** 2020-07-17

**Authors:** Yongfa Sun, Lili Song, Yong Zhang, Hongjun Wang, Xiao Dong

**Affiliations:** 1 College of Life Science, Qingdao Agricultural University, No. 700, Changcheng Road, Chengyang District, Qingdao, 266109 Shandong People’s Republic of China; 2 Medical University of South Carolina, Charleston, SC 29425 USA

**Keywords:** Mesenchymal stem cells, Angiogenesis, Diabetes, Wound healing, Macrophages

## Abstract

**Background:**

Diabetic patients suffer from impaired wound healing. Mesenchymal stem cell (MSC) therapy represents a promising approach toward improving skin wound healing through the release of soluble growth factors and cytokines that stimulate new vessel formation and modulate inflammation. Whether adipose tissue-derived MSCs (ASCs) from type 2 diabetes (T2D) donors are suitable for skin damage repair remains largely unknown.

**Methods:**

In this study, we compared the phenotype and functionality of ASCs harvested from high-fat diet (HFD) and streptozotocin (STZ)-induced T2D or control mice, and assessed their abilities to promote wound healing in an excisional wound splinting mouse model with T2D.

**Results:**

T2D ASCs expressed similar cellular markers as control ASCs but secreted less hepatocyte growth factor (HGF), vascular endothelial growth factor (VEGF), and transforming growth factor β (TGF-β). T2D ASCs were somewhat less effective in promoting healing of the wound, as manifested by slightly reduced re-epithelialization, cutaneous appendage regeneration, and collagen III deposition in wound tissues. In vitro, T2D ASCs promoted proliferation and migration of skin fibroblasts to a comparable extent as control ASCs via suppression of inflammation and macrophage infiltration.

**Conclusions:**

From these findings, we conclude that, although ASCs from T2D mice are marginally inferior to control ASCs, they possess comparable therapeutic effects in wound healing.

## Introduction

Type 2 diabetes (T2D) is a metabolic disease characterized by insulin resistance and pancreatic β cell dysfunction, which results in long-term hyperglycemia and various degenerative complications [1]. Insulin sensitizers and insulin injection are helpful in relieving hyperglycemia but are less effective in relieving symptoms of diabetes complications [2]. Improvements have been made in preventing morbidity and disability associated with diabetes complications in the past few decades [3]. Diabetic patients often suffer from impaired wound healing [4, 5], and approximately 5–8% of T2D patients develop chronic foot ulcers due to complications of diabetes [6]. Potential factors contributing to impaired wound healing in diabetes include sustained chronic inflammation, decreased secretion of growth factors, and disrupted vascularization [7].

MSCs are adult stem cells that can be harvested from multiple tissues including bone marrow, adipose tissue, and umbilical cord [8]. MSCs have low immunogenicity and rarely induce an immune response [9]. The differentiation and proliferation of MSCs at the site of a wound provide a favorable environment for tissue regeneration [10]. Transplantation of MSCs alone or in combination with biological scaffolds significantly promotes wound healing [10–13]. MSCs from various sources have been shown to effectively promote wound healing. For example, implantation of bone marrow-derived MSCs (BM-MSCs) enhanced wound healing in mice [14, 15], and transplantation of placenta-derived MSCs accelerated murine dermal wound closure [16].

The effects of adipose-derived MSCs (ASCs) in promoting wound healing have also been studied in animal models [17]. ASCs can self-renew and are as expandable as BM-MSCs. Comparative analysis of BM-MSCs and ASCs showed that they exhibit similar morphology, cell surface marker profiles, and differentiation abilities [18, 19]. ASCs are far more prevalent than BM-MSCs, and much larger numbers of ASCs can be readily harvested. Only 0.001–0.1% of the total nucleated cells in the bone marrow are MSCs [20], whereas approximately 1–10% of stroma-vascular fraction cells are MSCs [21–23]. ASCs in combination with Exendin-4 promoted angiogenesis and improved wound healing in C57BL/6 or leptin receptor-deficient (db/db) mice [24]. Infusion of human ASCs promoted repair of diabetic foot ulcers in rats [25]. ASCs in combination with light therapy enhanced angiogenesis and skin wound healing in mice [10]. Human ASCs seeded on a silk fibroin-chitosan scaffold enhanced wound repair by improving neovascularization in a murine soft tissue injury model [26]. Autologous human adipose tissue-derived follicle stem cells promoted human hair growth in androgenetic alopecia and improved face scar correction [27–29], [30]. These previous animal studies and clinical trials have assessed the therapeutic effects of healthy ASCs in wound healing in healthy mice or patients. However, diabetic patients suffer from impaired wound healing due to sustained chronic inflammation, decreased secretion of growth factors, and disrupted vascularization. Their disease conditions may reduce the therapeutic capability of ASCs.

In this study, we compared the phenotypes of ASCs harvested from HFD and STZ-induced T2D and healthy Chow diet-fed C57BL/6 mice in vitro and their therapeutic effects for the treatment of wound healing in a mouse model of T2D. A systemic evaluation of the therapeutic effects of T2D ASCs in the treatment of chronic inflammation, growth factor secretion, and revascularization in the T2D mouse wound healing models may facilitate our understanding of the mechanisms and potential clinical applications of using autologous ASCs to promote wound healing in diabetes patients.

## Materials and methods

### Animals

Four-week-old male C57BL/6J mice were purchased from the Vital River Laboratory Animal Technology Co., Ltd. (Beijing, People’s Republic of China). Mice were allowed to adapt to the new environment for 1 week and were then randomly divided into a HFD group (60% of calories from fat) or a standard control fat diet (Chow) control group (10% of calories from fat). Body weights and blood glucose levels of mice were measured weekly. At 16 weeks of age, the HFD mice were given one dose of STZ (Sigma-Aldrich, St. Louis, MO, USA) (40 mg/kg) to destroy pancreatic β cells. Intraperitoneal glucose tolerance test (IPGTT) and insulin tolerance test (ITT) were performed 2 weeks after STZ injection as previously described [31]. All animal experiments were approved by the Institutional Animal Care and Use Committee at Qingdao Agricultural University.

### Isolation and culture of ASCs

ASCs were isolated and cultured according to previously published protocols [31]. In brief, the epididymal fat pad was collected. Connective tissue was carefully dissected away under a dissecting microscope. Fat was washed with PBS, finely minced, and digested at 37 °C in PBS containing 0.25% collagenase type 1 (Sigma-Aldrich, St. Louis, MO, USA) for 45 min with agitation, and then centrifuged at 500*g* for 5 min to remove floating mature adipocytes. The cell suspension was filtered through a 200-μm cell strainer, and isolated cells were cultured in Dulbecco’s modified Eagle’s medium (DMEM) supplemented with 10% fetal bovine serum (FBS) and 1% penicillin-streptomycin (Shenggong Co., Ltd., Shanghai, China) at 37 °C, 5% CO_2_. After overnight incubation, non-adherent cells were removed, and the medium was replaced with fresh complete medium. To obtain ASC-conditioned medium, the medium was collected from non-confluent cells cultured for 3 days in a medium containing 2% FBS. The supernatant was centrifuged at 500*g* for 10 min to remove cell debris, was snap frozen, and was stored at − 80 °C until used.

### Characterization of ASCs

#### Flow cytometry

Specific cellular markers in ASCs were analyzed by flow cytometry as described [31]. In brief, ASCs were incubated with fluorescein isothiocyanate-conjugated rat anti-mouse monoclonal antibodies against CD34 or CD45, and phycoerythrin-conjugated antibodies against CD29 or CD105 before flow cytometry analysis. For each sample, a minimum of 100,000 cells was analyzed using a BD Flow Cytometer, and data were analyzed using the CellQuest software and displayed as histograms. The events were acquired and analyzed under the same conditions; cell debris was excluded from the analysis. Cell surface marker expression was determined by comparison with corresponding isotype controls.

#### Differentiation

ASCs were induced to differentiate into adipocytes, osteoblasts, or chondrocytes using cell differentiation kits according to the manufacturer’s recommendations (Mo Bi Tec. GmbH, Lorzestrasse, Germany). The presence of adipocytes was determined by Oil Red O staining, osteoblasts by Alizarin Red staining, and chondrocytes by Alkaline Blue staining as described previously [31]**.**

#### Cell proliferation

Rates of proliferation of ASCs were measured by XTT Cell Proliferation Assay kits according to the manufacturer’s instructions (ATCC). ASCs were dispersed into 100 μl of cell suspension in a 96-well plate (2000 cells/well) and cultured overnight before the addition of 10 μl cell counting solution. Cells were incubated for 1 h, and absorbance at 450 nm was measured using a microplate reader. Cells were counted daily for 7 days.

### Generation of mouse wound model and ASC infusion

Mice were anesthetized with ketamine, the hair was removed from the dorsal surface, and two 5-mm full thickness excisional skin wounds were created on each side of the midline. To inhibit wound contraction, a 0.5-mm-thick silicone splint was applied over the wound. T2D mice were divided into three treatment groups of 12 mice per group: (i) T2D control which was injected with PBS only, (ii) T2D mice receiving ASCs from healthy C57BL/6 mice, and (iii) T2D mice receiving ASCs from T2D mice (T2D ASCs). C57BL/6J mice fed normal chow injected with PBS were used as healthy controls (Chow). In mice receiving ASCs, 5 × 10^5^ ASCs re-suspended in 200 μl of PBS were injected locally around the excisional wounds.

Digital photographs of wounds from each mouse were taken daily. The wound margin and the wound area were calculated using the ImageJ software. Percent closure of wounds was calculated using the following formula: percent closure = {(area of original wound − area of actual wound)/area of original wound} × 100.

### Hematoxylin and eosin staining

The granulation tissue and surrounding skin tissues were removed and fixed in 4% paraformaldehyde, gradually dehydrated, embedded in paraffin, and cut into 5-μm sections. A total of 60 sections were collected from each wound at 50 μm intervals. Tissue sections were stained in hematoxylin for 10 min, rinsed with water, and then stained with eosin for 1 min. H&E-stained slides were observed under an Olympus BX51 microscope (Olympus, Tokyo, Japan).

### Immunohistochemistry staining

Capillary density in skin tissue was assessed morphometrically by examining four random fields of equal size per section of wound excluding wound edges in six successive sections after immunofluorescence staining for endothelial cells with an anti-CD34 antibody (1:100, Abcam, Cambridge, MA, USA). Macrophages were identified by staining with an F4/80 antibody (1:100, Abcam, Cambridge, MA, USA). For immunofluorescence staining, tissue sections were treated with antigen retrieval solution at 95 °C for 10 min after rehydration and then with 3% hydrogen peroxide to inactivate endogenous peroxidases. Tissue sections were incubated with the primary antibody for 24 h at 4 °C and then with horseradish peroxidase-conjugated secondary antibody at 37 °C for 45 min. Sections were washed, and signals were observed under a fluorescence microscope.

### Real time PCR analysis

Total RNA was extracted from wound granulation tissue using an RNA extraction kit (Aidlab Biotechnologies Co., Ltd., Beijing, China). RNA was transcribed into cDNA using reverse transcriptase. The following primer pairs were used: interleukin-1β (IL-1β)—5′ GAGCACCTTCTTTTCCTTC, 3′ GTTCATCTCGGAGCCTGTA; tumor necrosis factor α (TNF-α)—5′ TTCTCAAAATTCGAGTGAC, 3′ TAGACAAGGTACAACCCAT; IL-10—5′ CCAAGCCTTATCGGAAATGA, 3′ TCCTGAGGGTCTTCAGCTTC; transforming growth factor β (TGF-β)—5′ ACTTGCAAAGGGCTCTGGTA, 3′ AATGGCTTCCACCCTCTTCT; and vascular endothelial growth factor (VEGF)—5′ ATCTTCAAGCCGTCCTGTGT, 3′ AGGTTTGATCCGCATGATCT. β-Actin expression was quantified in each sample and used as an endogenous control.

### Western blot

Total tissue lysates were extracted in a lysis buffer containing 1% Triton X-100 and proteinase inhibitors (Shanghai Shenggong Ltd., Shanghai, China). Total proteins were separated on a 6% SDS-polyacrylamide gel and transferred to nitrocellulose membranes. Membranes were incubated with a monoclonal antibody against collagen (1:5000, Abcam, Cambridge, MA, USA) overnight at 4 °C. The membrane was then washed and incubated with the secondary antibody (HRP-conjugated goat anti-rabbit polyclonal antibody, 1:1000; Abcam, Cambridge, MA, USA) for 2 h at room temperature, and signal was observed after incubation with the ECL Western blot substrate.

### Enzyme-linked immunosorbent assay

Concentrations of VEGF, HGF, and TGF-β in ASC-conditioned medium were measured with specific ELISA kits based on the manufacturer’s recommendations (Gudo, Shanghai CO., Ltd., China). In brief, the culture media were diluted using assay diluent, added into individual wells pre-coated with the specific capturing antibodies, and incubated for 1–2 h at 37 °C. The remaining protein binding sites were blocked in blocking buffer (5% non-fat milk in PBS) and then incubated with HRP-conjugated antibody at room temperature and then with substrate solution for signal detection. Color intensity was measured using a microplate reader at the OD 450.

### Isolation of skin fibroblasts and co-culture with ASCs

Skin tissues of neonatal C57BL/6 mice were collected, washed with PBS, finely minced, and digested at 37 °C in PBS containing 0.25% collagenase I (Shenggong Biotech Co., Ltd., Shanghai, China) for 45 min with agitation. Isolated cells were cultured with complete medium. Non-adherent cells were discarded, and the medium was replaced after overnight incubation. Cell proliferation in the presence or absence of ASCs was determined using a Transwell system. Fibroblasts (1 × 10^4^ cells/well) were seeded into the bottom wells. ASCs (1 × 10 ^4^ cells/well) were seeded into the upper wells. Fibroblasts were counted daily until 3 days post-co-culture.

To measure fibroblast migration, a “wound” was generated in a confluent fibroblast culture using a pipet tip, and cell debris was removed by PBS washing. Cells co-cultured with medium alone were used as the negative control. Migration of fibroblast into to the “wound” area was observed at 10 and 20 h after wounding. Images of two different areas of equal size per culture were acquired using a digital camera. The residual area between fibroblasts was determined by computer-assisted image analysis using the ImageJ software, and the percent of wound area remaining compared to the initial scratched area was calculated.

### Statistical analysis

Each experiment was performed independently at least three times. Data are presented as mean ± standard derivation (SD). Student’s *t* test or ANOVA tests were performed to compare the differences between samples. Differences were considered statistically significant for two-tailed values of *P* < 0.05.

## Results

### Generation of the T2D mouse model

**Fig. 1:**
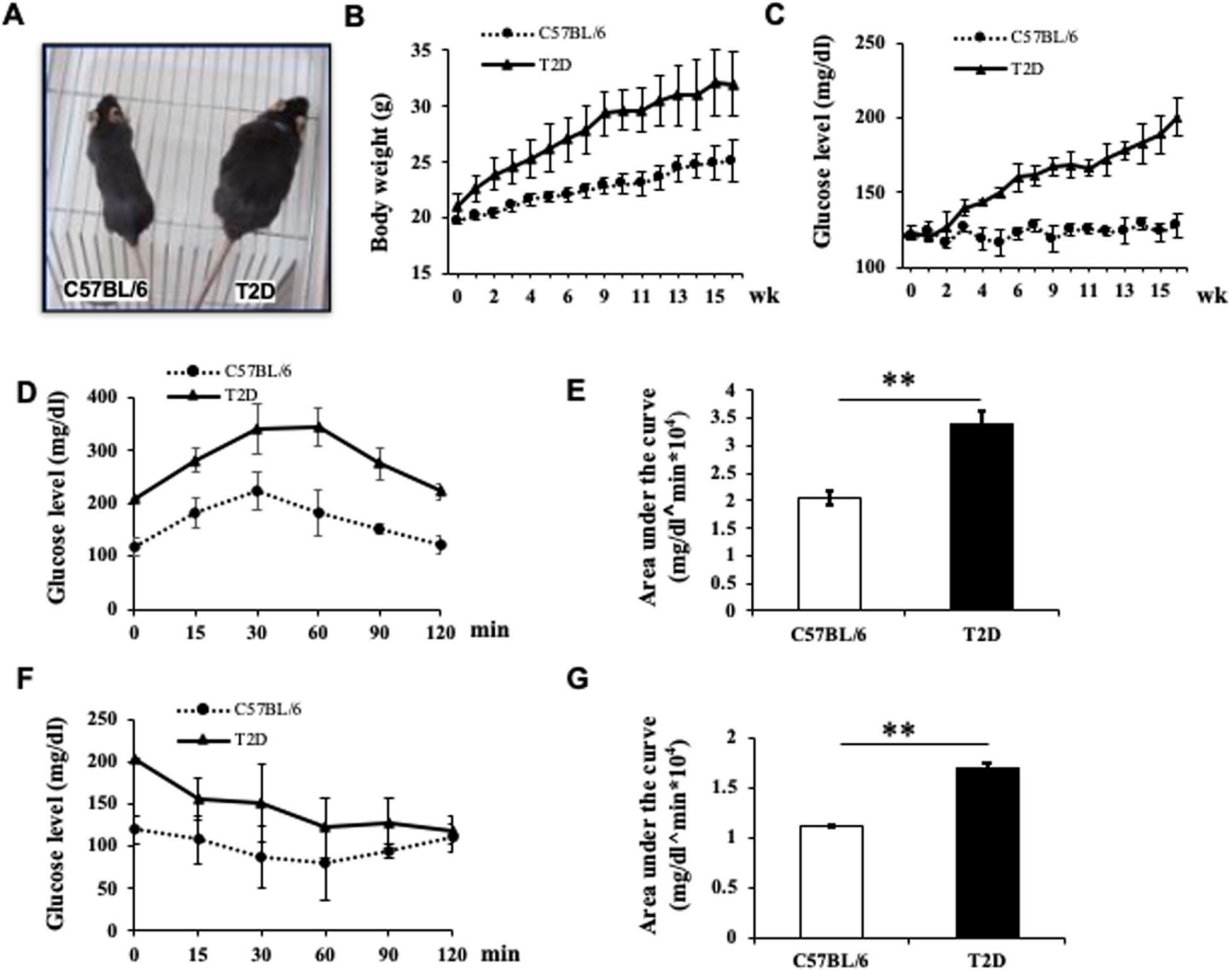
Generation of T2D mice by high-fat diet feeding and STZ injection. **a** Image of representative T2D and C57BL/6 control mice at 19 weeks of age. **b** Body weights and **c** fed blood glucose levels of T2D mice and Chow diet mice (Chow). **d** Blood glucose levels of Chow and T2D mice during an IPGTT. **e** Area under the curve of IPGTT. **f** Blood glucose levels during the ITT of T2D mice and C57BL/6 mice. **g** Area under the curve during the ITT. C57BL/6 group, *n* = 24, T2D group, *n* = 46. *P < 0.05; **P < 0.01, ANOVA test. T2D mice: fed high-fat diet and received STZ injection; C57BL/6: mice fed standard diet

We generated the T2D mouse model by continuous HFD feeding of C57BL/6 mice in combination with STZ injection. Body weights and blood glucose levels were significantly higher in HFD mice than C57BL/6 (or Chow) mice after 11 weeks of HFD feeding, at which time STZ (40 mg/kg) was given to HFD mice to induce islet cell death. At 16 weeks, the average body weight of STZ-treated HFD mice was 31.99 ± 1.87 g (*n* = 42), compared with 25.10 ± 2.84 g for Chow mice (*n* = 24, Fig. 1a, b). The average blood glucose level in STZ-HFD mice was 210 ± 21 mg/dl, compared with 124 ± 6 mg/dl in controls (Fig. 1c). Furthermore, STZ-treated HFD mice showed impaired glucose disposal in the IPGTT test (Fig. 1d, e) and reduced insulin sensitivity in the ITT test (Fig. 1f, g). STZ-treated HFD mice with insulin resistance and blood glucose levels > 200 mg/dl were considered T2D mice.

**Fig. 2:**
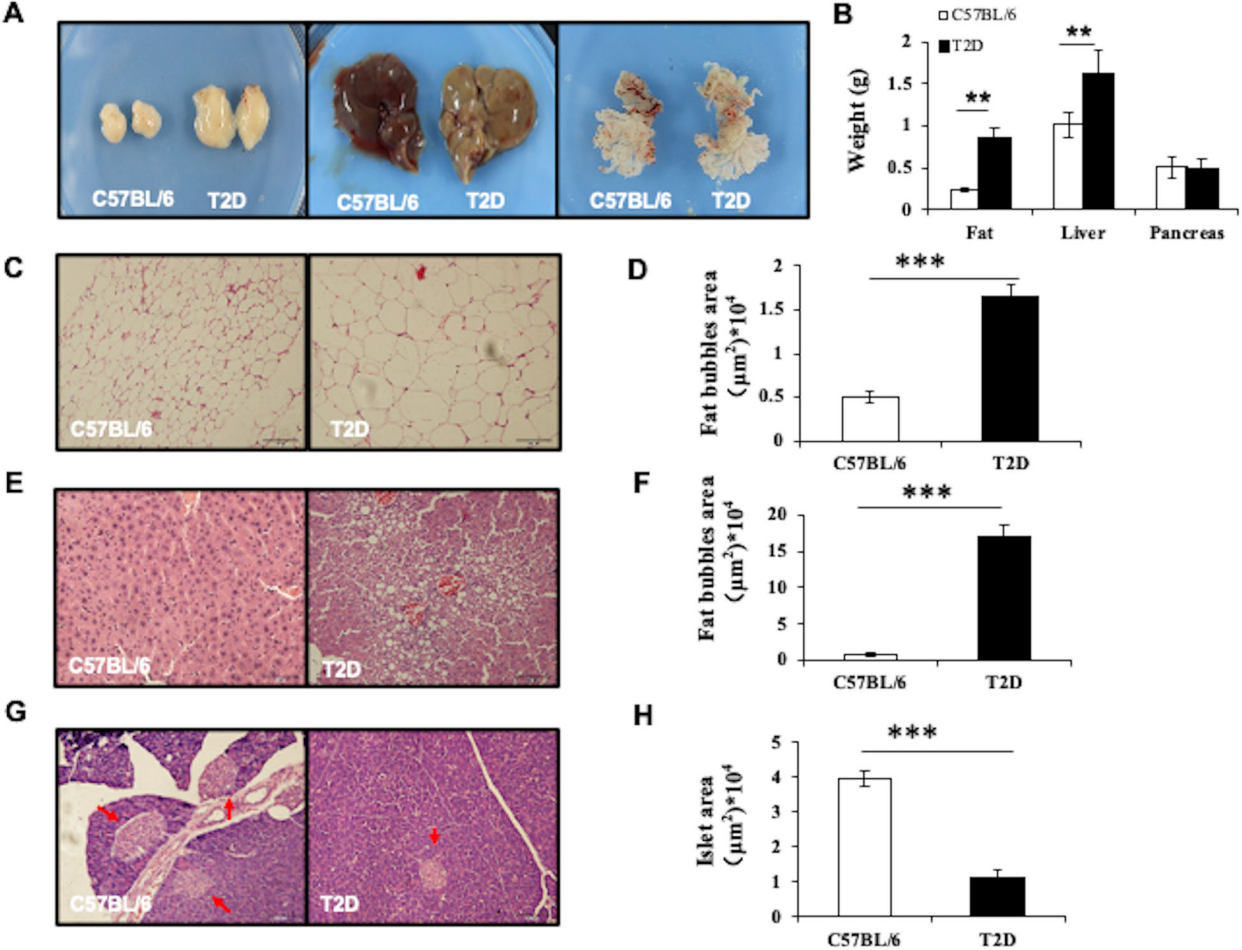
Changes in the fat, liver, and pancreases in T2D mice. **a** Micrographs of the fat, liver, and pancreas from age-matched C57BL/6 control and T2D mice. **b** Weights of the fat, liver, and pancreas in C57BL/6 and T2D mice. **c**, **e**, **g** H&E stain of the fat tissue, liver, and pancreas of C57BL/6 and T2D mice. **d** Average size of the fat bubble in adipose tissue. **f** Quantification of fat bubble size in liver tissue. **h** Quantification islet area within each pancreas tissue. At least 5 mice were included in each group. *P < 0.05; **P < 0.01, ANOVA test

We further characterized the T2D mice. T2D mice had increased fat and liver weights compared to Chow mice (*n* = 5 in each group, Fig. 2a, b). The average tissue weights in the T2D mice were 0.86 ± 0.36 g for adipose tissue and 1.61 ± 0.29 g for liver, compared to 0.24 ± 0.03 g and 1.02 ± 0.15 g, respectively, in controls (*P* < 0.05) (Fig. 2a). H&E staining showed that T2D mice had enlarged adipocytes in the epididymal fat, with an average fat bubble size of 16,579 μm^2^ compared to 5002 μm^2^ in controls (Fig. 2c, d). The T2D mice exhibited liver steatosis with adipocytes occupying an area of approximately 170,000 μm^2^, compared to 7000 μm^2^ in controls (Fig. 2e, f**)**. T2D mice showed a dramatic decrease in the islet area with an average of 11,402 μm^2^ in T2D compared to 39,517 μm^2^ in controls (Fig. 2g, h). These data are comparable to other HFD and STZ-induced T2D mouse models characterized by insulin resistance, enlarged adipocytes, liver steatosis, and reduced pancreatic islet mass.

### Characterization of ASCs harvested from T2D or control mice

**Fig. 3:**
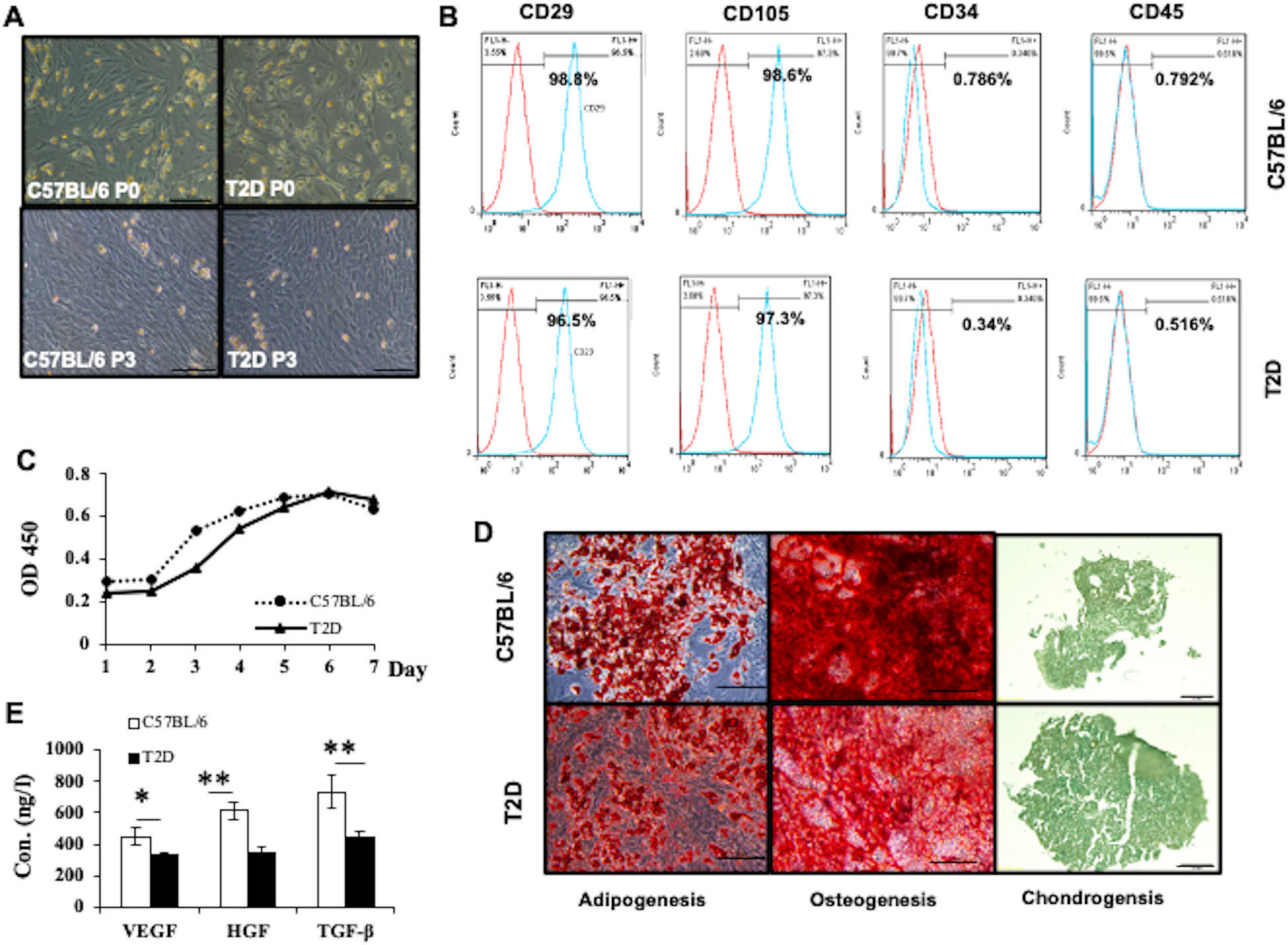
Characterization of ASCs isolated from C57BL/6 and T2D mice. **a** Representative micrographs of C57BL/6 and T2D ASCs at passage 0 and passage 3 observed under a light microscope. **b** Expression of CD29, CD105, CD34, and CD45 in ASCs harvested from C57BL/6L or T2D mice analyzed by flow cytometry. **c** Growth curves of C57BL/6 and T2D ASCs at passage 3. **d** The morphology of adipocytes, osteocytes, and chondrocytes derived from C57BL/6 ASCs and T2D ASCs identified by Oil Red, Alizarin Red, and Alcian Blue staining, respectively. Scale bar = 100 μm. **e** Concentrations of VEGF, HGF, and TGF-β secreted by C57BL/6 ASCs or T2D ASCs. Data are mean ± SEM of at least three individual experiments. At least 3 mice were included in each group. *P < 0.05, **P < 0.01, ANOVA test

We compared the phenotypes of ASCs harvested from T2D or Chow mice (*n* = 3). Cells of both origins showed similar morphology (Fig. 3a). They both had highly expressed CD105 (> 95%) and CD29 (> 95%), and low expression of CD34 (< 5%) and CD45 (< 5%) (Fig. 3b). Although the proliferation rate was not significantly different, T2D ASCs proliferated somewhat more slowly than control ASCs at passage 3 (Fig. 3c). They both differentiate into adipocytes, chondrocytes, and osteocytes (Fig. 3d). However, T2D ASCs secreted significantly less VEGF, HGF, and TGF-β than Chow ASCs (Fig. 3e). These data show that ASCs from T2D mice share similar phenotypes with ASCs from healthy mice, albeit with reduced cytokine production.

### The effects of ASCs on wound healing

**Fig. 4:**
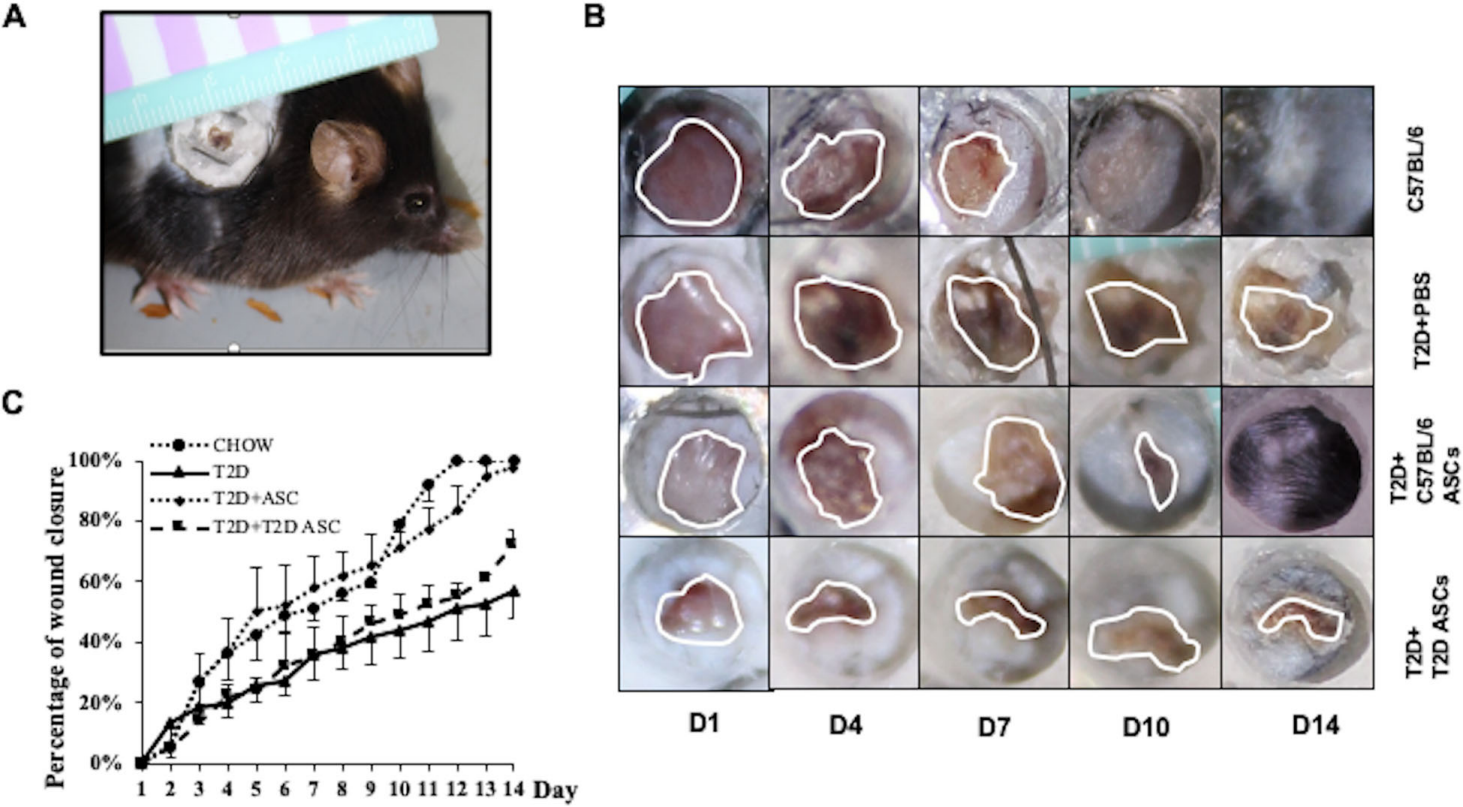
Infusion of ASCs promotes wound healing. **a** Splinted excisional mouse wound model. **b** Images of wound photographed at 1 to 14 days post-wounding in Chow or T2D mice receiving PBS, C57BL/6 ASCs, or T2D ASCs. **c** Percentage of healing areas calculated from images using the ImageJ software. At least 3 mice were used in each group

We next determined the effects of the two types of ASCs on wound healing using the excisional wound-splinting model in T2D mice (Fig. 4a). Unlike human wounds, contraction is the main mechanism of mouse skin wound closure [32]. To mimic the healing process of human wounds, we placed a silicone splint around the wound to prevent skin contraction. Wounds were made in T2D mice and C57BL/6 mice (healthy controls). Then, T2D ASCs or Chow ASCs (1 × 10^5^/mouse) or PBS (control) were injected locally around the wound, and wound closure rate was measured. In PBS controls, T2D mice showed significantly slower wound closure than the healthy controls, i.e., 100% of wounds were completely closed in the control mice at 14 days post-wounding, whereas, only 56.67% of wounds were closed in T2D mice receiving PBS at 14 days post-wounding. In T2D mice receiving Chow ASCs, 83% of wounds had healed, whereas in T2D mice receiving T2D ASCs, 72.32% of wounds had healed at 14 days post-wounding (Fig. 4b, c). These data show that both healthy ASCs and T2D ASCs promote wound healing in T2D mice.

### The effect of ASCs on skin regeneration

**Fig. 5:**
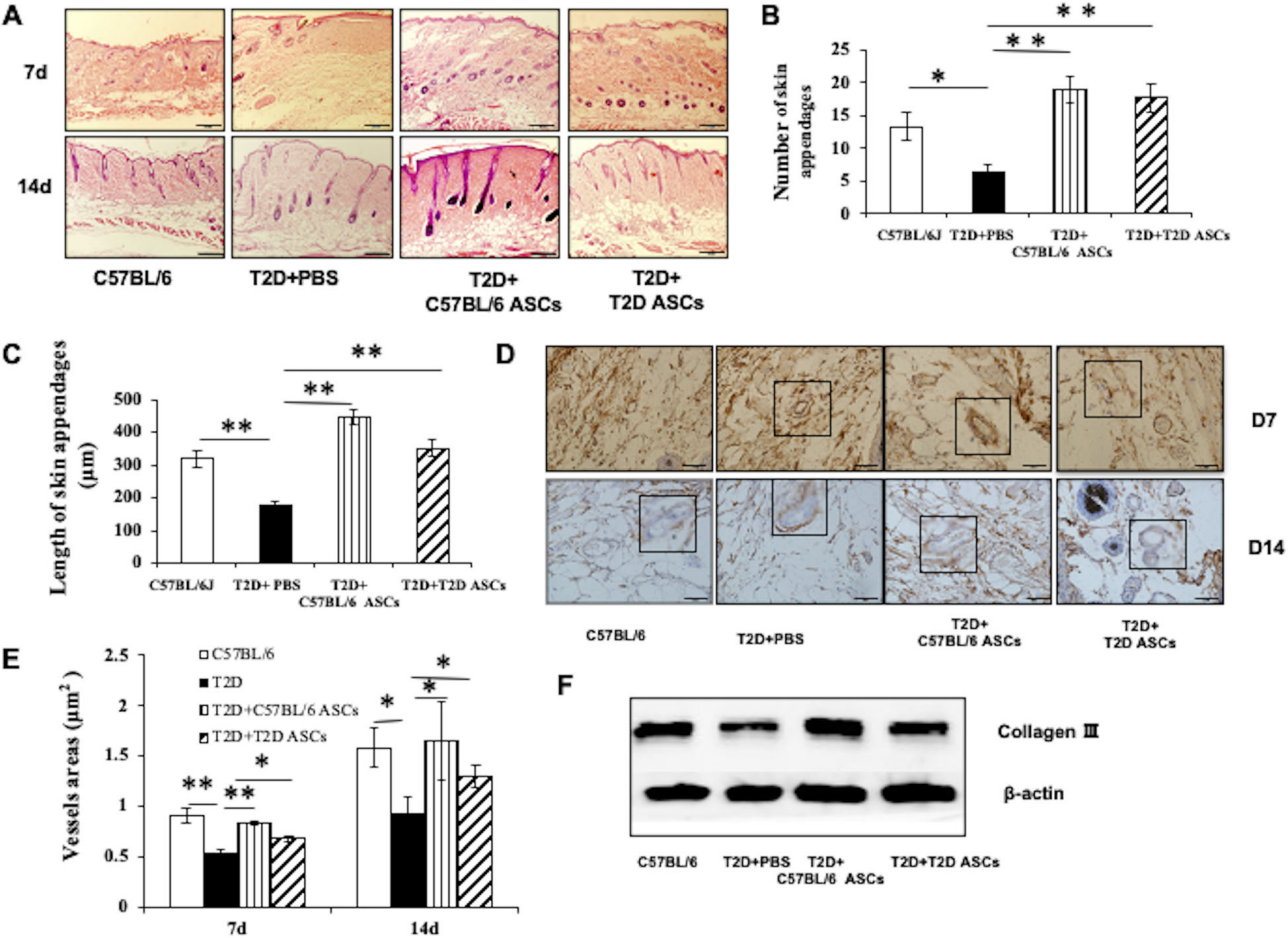
The effect of ASCs on granulation tissue formation. **a** H&E staining at day 7 and 14 post-wounding in Chow or T2D mice receiving PBS, C57BL/6 ASCs, or T2D ASCs. **b** The number of epidermal sleeves in mice from different groups. **c** The length of epidermal sleeves in mice at 14 days post-wounding. **d** Immunohistochemical staining of CD34^+^ cells in wound tissue at 7 and 14 days post-treatment. **e** Vessel areas in mice from different groups at 7 and 14 days post-treatment. **f** Deposition of collagen III in granulation tissue of 2 groups of mice. T2D: mice fed high-fat diet receiving STZ injection; C57BL/6: mice fed standard diet. At least 3 mice were used in each group. *P < 0.05, **P < 0.01, ANOVA test

We next assessed the effect of ASCs on skin regeneration by measuring numbers and lengths newly formed epidermal sleeves. At 14 days post-treatment, the average number of epidermal sleeves was 6.33 ± 0.58 in T2D mice receiving PBS, 18.67 ± 1.53 in T2D mice receiving Chow ASCs, and 17.33 ± 1.53 in T2D mice receiving T2D ASCs (Fig. 5a, b). The length of epidermal sleeves was 167.78 ± 13.67 μm in T2D mice receiving PBS, 454.78 ± 21.49 μm in T2D mice receiving Chow ASCs, and 358.67 ± 26.63 μm in T2D mice receiving T2D ASCs (Fig. 5a, c). Therefore, treatment with ASCs improves re-epithelialization, seen as increased numbers and lengths of epidermal sleeves.

Angiogenesis is critical for wound healing [33]. The formation of new blood vessels can provide nutrients to support cell regeneration. To determine whether local ASC injection contributed to angiogenesis, we measured vessel area by staining for CD34^+^ endothelial cells in skin tissue sections at 7 and 14 days post-treatment. We found that at 14 days post-treatment, the average vessel area was 9222 ± 1654 μm^2^ in T2D mice receiving PBS, 16,535 ± 3875 μm^2^ in T2D mice receiving Chow ASCs, and 12,909 ± 1147 μm^2^ in T2D mice receiving T2D ASCs (Fig. 5d, e). We also measured the expression levels of collagen III by Western blot analysis. Collagen III is an abundant extracellular matrix protein in skin tissue, which contributes to wound healing. Our data showed that treatment with either Chow ASCs or T2D ASCs promoted collagen III deposition in wounded skin tissue (Fig. 5f), which subsequently contributed to wound healing.

### ASC treatment suppresses macrophage infiltration and inflammation

**Fig. 6:**
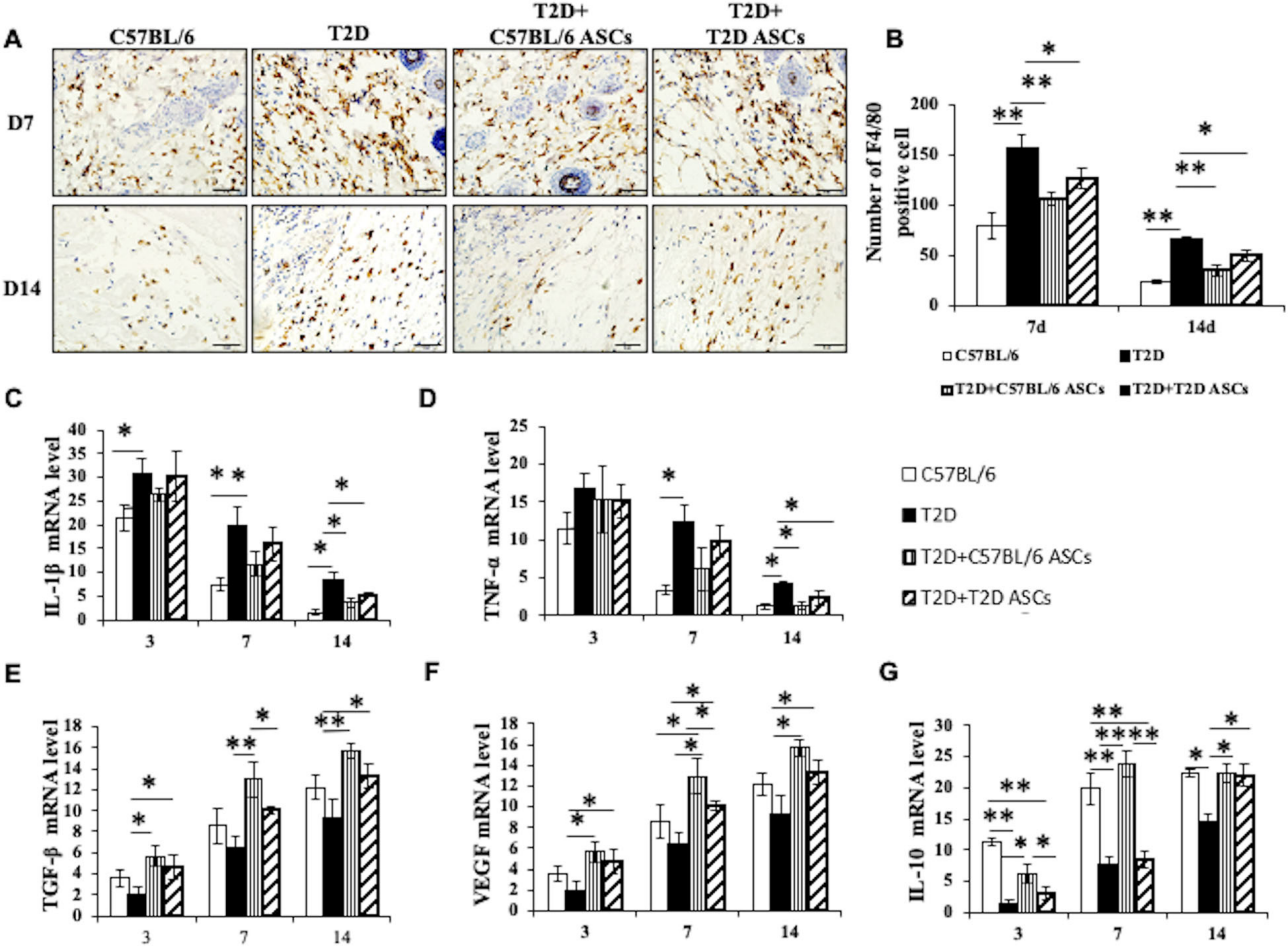
ASCs regulate macrophages and inflammatory reaction. **a** Immunohistochemical staining of F4/80+ cells in wounds from Chow or T2D mice treated with PBS, C57BL/6 ASCs, or T2D ASCs. **b** The number of macrophages in mice at 7 and 14 days post-treatment. **c** mRNA levels of pro-inflammatory response IL-1 and **d** TNF-α in wounds and **e** relative mRNA levels of TGF-β, **f** VEGF, and **g** IL-10 in wound tissues; *n* = 3 mice per group. *P < 0.05, **P < 0.01, ANOVA test

We next examined the presence of macrophages, critical players in inflammation, in wound tissues at 7 and 14 days after ASC treatment. We found that, at both time points, granulation tissue in T2D mice receiving T2D ASCs exhibited greater macrophage infiltration than granulation tissue in T2D mice receiving Chow ASCs (Fig. 6a). The mean numbers of F4/80+ macrophages in granulation tissue was 156 ± 14 at day 7 and 65 ± 4 at day 14 in T2D mice receiving PBS, 107 ± 6 at day 7 and 35 ± 5 at day 14 in T2D mice receiving Chow ASCs, and 127 ± 11 at day 7 and 50 ± 5 at day 14 in T2D mice receiving T2D ASCs (Fig. 6a, b). Consistent with the macrophage levels, the expression of macrophage-associated genes including IL-1β (Fig. 6c) and TNF-α (Fig. 6d) were high at day 7 but significantly reduced at 14 days after wounding. Expression levels of IL-1β and TNF-α in T2D mice receiving PBS remained at a high level from day 3 to day 7 after wounding. However, IL-1β and TNF-α levels were significantly lower in T2D mice receiving T2D ASCs or Chow ASCs. Concomitantly, the expression of the anti-inflammatory genes TGF-β (Fig. 6e), VEGF (Fig. 6f), and IL-10 (Fig. 6g) increased from day 3 to day 7 and day 14 after wounding. The expression of TGF-β (Fig. 6e), VEGF (Fig. 6f), and IL-10 (Fig. 6g) in T2D mice receiving PBS were low at day 14 compared with T2D mice receiving T2D ASCs. These data show that ASC infusion reduced numbers of macrophages and expression of pro-inflammatory cytokines and increased expression of protective molecules that might contribute to accelerated wound healing.

### ASCs promote migration and proliferation of fibroblasts in vitro

**Fig. 7:**
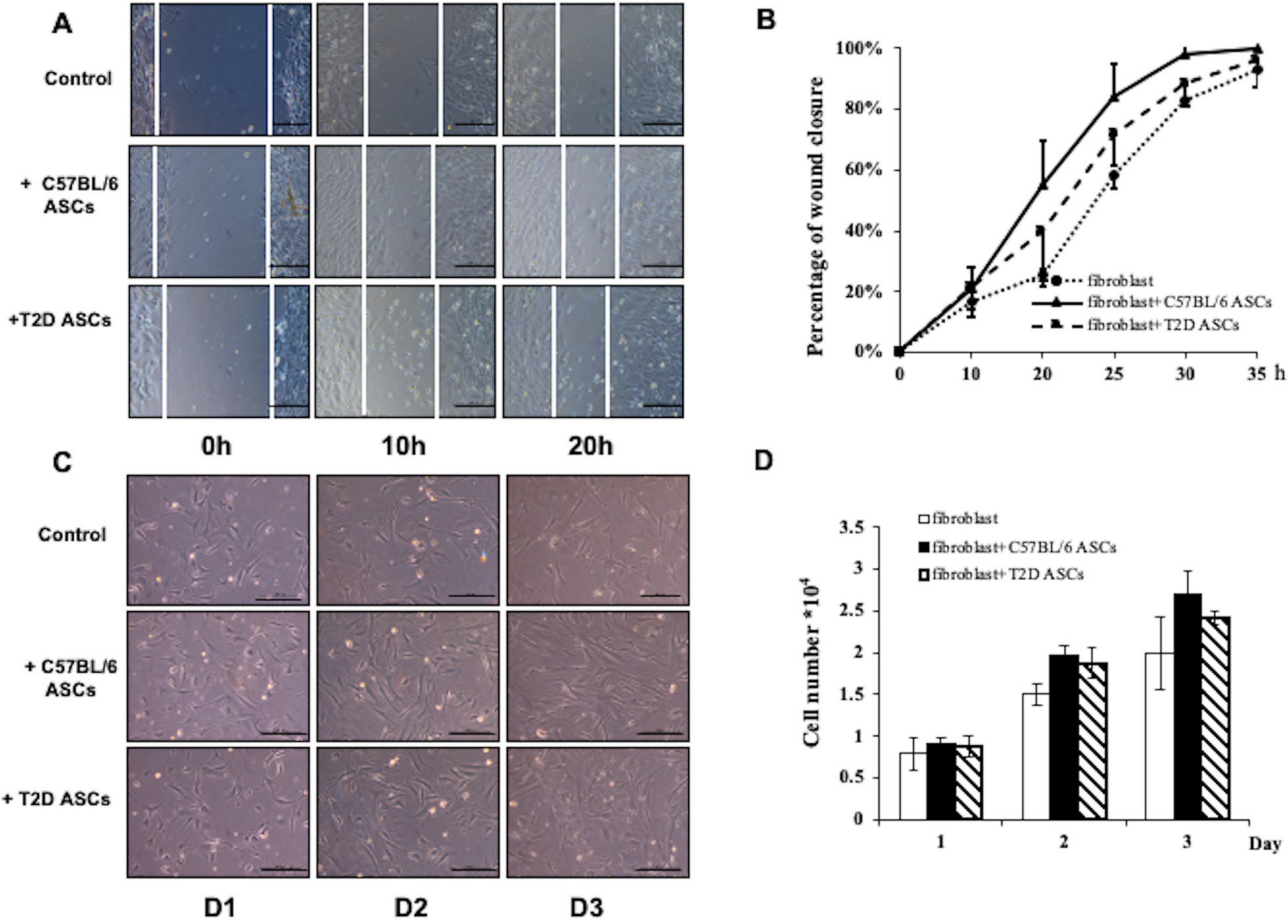
ASCs promote fibroblast migration and proliferation in vitro. **a** Image of fibroblasts migrating into the scratched area in fibroblast cultures. Fibroblasts were cultured alone or co-cultured with C57BL/6L ASCs or T2D ASCs. **b** The area covered by fibroblasts in each group. **c** Representative image of fibroblast proliferation from each group. **d** The numbers of fibroblasts in each group at 1, 2, and 3 days post-wounding. Data are representative of at least 3 individual experiments. At least 3 mice were used in each group. *P < 0.05, **P < 0.01, ANOVA test

Granulation tissue plays an essential role in wound healing by providing scaffolds for the assembly of neighboring cells at wound margins. Fibroblasts are major cell types in the granulation tissue and can rapidly proliferate and migrate to the wound site. MSCs can promote proliferation, migration, and collagen secretion of fibroblasts through a paracrine mechanism. We compared the ability of Chow ASCs and T2D ASCs to promote the migration of mouse fibroblasts to an “injured” area in vitro using a standard Transwell system in which fibroblasts were co-cultured with or without ASCs [34]. At 20 h post-wound generation, 58 ± 4% of the wound area in the control cells not co-cultured with ASCs was covered by monolayer adherent fibroblasts. In wells co-cultured with Chow ASCs, 84 ± 15% of the wound area was covered, and in wells co-cultured with T2D ASCs, 72 ± 2% of the wound area was covered (Fig. 7a, b). Further studies showed that co-culture with ASCs improved proliferation of fibroblasts, although the differences did not reach significance (Fig. 7c, d). These data show that Chow ASCs and T2D ASCs promoted the migration of fibroblasts in vitro.

## Discussion

In this study, we assessed the potential role of ASCs harvested from T2D mice in wound healing in the T2D mouse models. Our most dramatic finding was that T2D ASCs were effective in promoting wound healing, although the efficiency was lower than ASCs harvested from healthy, age-matched control mice. We found that T2D ASCs shared similar phenotypes and differentiation abilities as healthy ASCs. However, T2D ASCs secreted lower amount of VEGF, HGF, and TGF-β, three major cytokines that mediate the protective effects of ASCs, which might have affected the therapeutic effect of T2D ASCs in wound healing.

Diabetic patients often suffer from both microvascular and macrovascular disease caused by ischemia and hypoxia [35–37]. A long-term hyperglycemic microenvironment in diabetes leads to microvascular cell loss and blood flow abnormalities. This environment may impact the therapeutic effects of ASCs. Indeed, in our study, the morphology of T2D ASCs was less replete compared to control ASCs at passage 0. We further compared the morphology and cell growth of T2D ASCs and control ASCs at passage 1 and passage 3. The shape of T2D ASCs was similar to the shape of control ASCs at passage 3, but growth was slower. The reduced growth of T2D ASCs might have been caused by hyperglycemia and inflammation under diabetic conditions. In contrast, the growth curves of ASCs at passage 3 showed that the proliferation rate of T2D ASCs was only slightly lower than control ASCs. We found no significant difference in the expression of cellular markers between ASCs harvested from controls or from T2D mice. Both T2D ASCs and control ASCs were positive for CD29 and CD105, and negative for CD45 and CD34, and could differentiate into chondrocytes, adipocytes, and osteocytes. These data differ in reports that T2D ASCs express lower levels of stem cell-specific markers [38] and fail to differentiate into functional adipocytes compared with control ASCs [39]. Differences in mouse strains used and disease severity might have contributed to this difference. However, T2D ASCs did secrete significantly lower amounts of VEGF, HGF, and TGF-β. All of these results suggest that the presence of diabetes had a dramatic impact on the phenotypes and characteristics of T2D ASCs.

The cutaneous wound healing process includes initiation, maintenance, and resolution phases [40–44]. Macrophages exhibit critical regulatory activities during these overlapping processes. During the initiation/inflammatory phase, inflammatory monocytes migrate to the injured tissues and differentiate into macrophages. The macrophages then participate in the elimination of microbial contaminants and mediate tissue repair, fibrosis regeneration, and prevention of infection. These macrophages produce high levels of pro-inflammatory cytokines including IL-1β and TNF-α. After the initial inflammatory phase subsides, the predominant macrophage population assumes a wound healing phenotype and secrete high levels of anti-inflammatory cytokines such as IL-10, TGF-β, and VEGF, which promote cellular proliferation and blood vessel development [45]. They also produce soluble mediators that stimulate local and recruited tissue fibroblasts to differentiate into myofibroblasts that facilitate wound contraction and closure, and secrete extracellular matrix components [46]. The transition of macrophages from an inflammatory to a protective phenotype plays an important role during the process of wound healing. Excessive secretion of pro-inflammatory cytokines including TNF-α and IL-1 β by macrophages at the wound site leads to chronic inflammation and represents a major characteristic feature of diabetic wounds [47].

We used an antibody that recognizes the mouse F4/80 antigen to identify macrophages in granulation tissue. In T2D mice receiving T2D ASCs, a significantly greater number of macrophages remained in the granulation tissue compared with T2D mice receiving Chow ASCs at the second week after treatment. T2D mice receiving Chow ASCs had accelerated disappearance of macrophages similar to Chow mice receiving Chow ASCs. In contrast, mice receiving T2D ASCs also had accelerated disappearance of macrophages, albeit to a slightly less extent than those receiving C57BL/6J ASCs, but suggesting that either source of ASCs reduced inflammation.

We observed changes in the expression of inflammatory cytokines in wound tissues in control mice and mice receiving ASCs. In control mice, TNF-α and IL-1β were highly expressed at the initial stage, while VEGF, IL-10, and TGF-β gradually increased at later stages of wound healing. Both C57BL/6J ASCs and T2D ASCs effectively suppressed the excessive expression of pro-inflammatory cytokines at the diabetic wound site. However, the IL-10 levels were low in mice receiving PBS or T2D ASCs at day 7, which may explain why mice receiving T2D ASCs exhibited delayed wound healing compared to mice receiving C57BL/6 ASCs.

Inflammation is accompanied by tissue regeneration, while the formation of granulation tissue requires cell proliferation and migration, deposition of extracellular matrix (ECM), and generation of blood vessels [48]. The deposition of ECM such as collagen III provides a scaffold for the migration of fibroblasts and the elongation of blood vessels [49, 50]. The regeneration of new blood vessels provides nutrients to fibroblasts [10]. As the main cell type of skin tissue, proliferation and migration of fibroblasts in ECM are the key factors promoting the formation of granulation tissue [5], which in turn secretes more ECM and growth factors that promote angiogenesis. In this study, ASCs promoted migration and proliferation of fibroblast when co-cultured with ASCs, and promoted the deposition of collagen III and neovascularization in granulation tissue in the T2D mouse wound model. T2D ASCs showed partially impaired protection, which may be caused by reduced secretion of VEGF, HGF, and TGF-β, which are critical for collagen synthesis and neovascular formation.

Similar to our studies, other studies have demonstrated that diabetes could cause damage to MSCs. For example, the survival and proliferation of MSCs are impaired by complications in individuals with T2D [51]. Persistent high glucose concentrations lead to cellular senescence and apoptosis, reduce proliferative capacity, and reduce osteogenic and chondrogenic potential of ASCs [52]. Selective depletion of BM-MSC subpopulations in different types of diabetes has an irreversible effect on their role in promoting new blood vessels [53]. Treating human MSCs with a serum derived from T2D patients decreases migration and trans-differentiation abilities of human MSCs [54]. Diabetes serum induces apoptosis of human BM-MSCs by the induction of autophagy signaling and decreases chemotaxis and their ability to differentiate into the endothelium [55]. The expression of early growth response factor-1 (EGR-1), PTEN, and GGPS1 are increased in ASCs from diabetic animals compared with C57BL/6J ASCs, which further reduce their effectiveness in promoting wound healing [56].

In conclusion, the therapeutic effects of T2D ASCs were reduced compared to healthy cells, and such reduction might have been caused by less secretion of cytokines that mediate the protection of ASCs including VEGF, HGF, and TGF-β. However, T2D ASCs exhibited potential to restore wound healing, suggesting their potential for use in autologous stem cell therapy in humans.

## Data Availability

The data used and/or analyzed during the current study are available from the corresponding author on reasonable request.

## Abbreviations

MSC: Mesenchymal stem cell
ASCs: Adipose-derived MSCs
HFD: High-fat diet
STZ: Streptozotocin
HGF: Hepatocyte growth factor
VEGF: Vascular endothelial growth factor
TGF-β: Transforming growth factor β
T2D: Type 2 diabetes
BM-MSCs: Bone marrow-derived MSCs
Chow: Control fat diet
IPGTT: Intraperitoneal glucose tolerance test
ITT: Insulin tolerance test
IL-1β: Interleukin-1β
TNF-α: Tumor necrosis factor α
