# Correction: Adipose stem cells from type 2 diabetic mice exhibit therapeutic potential in wound healing

**DOI:** 10.1186/s13287-022-02778-3

**Published:** 2022-02-25

**Authors:** Yongfa Sun, Lili Song, Yong Zhang, Hongjun Wang, Xiao Dong

**Affiliations:** 1 College of Life Science, Qingdao Agricultural University, No. 700, Changcheng Road, Chengyang District, Qingdao, 266109 Shandong People’s Republic of China; 2 Medical University of South Carolina, Charleston, SC 29425 USA

## Correction to: Stem Cell Research & Therapy (2020) 11:298 https://doi.org/10.1186/s13287-020-01817-1

The authors regret that they identified inadvertent errors during manuscript preparation that incorrect photos have been mis-uploaded in Fig. 3. Corrections made are listed below.

Figure 3B: The flow cytometry sub-figures in the low panels were duplicates of the upper panel instead of correct figures from the T2D group. It has now been replaced with the right sub-figures.

Figure 3D: C57BL/6 Adipogenesis (upper panel, left): A figure from the T2D group was mistakenly uploaded and is now corrected.

Figure 3D: Chondrogenesis: C57BL/6 and T2D subfigures were misplaced, and now corrected.

**Fig. 3:**
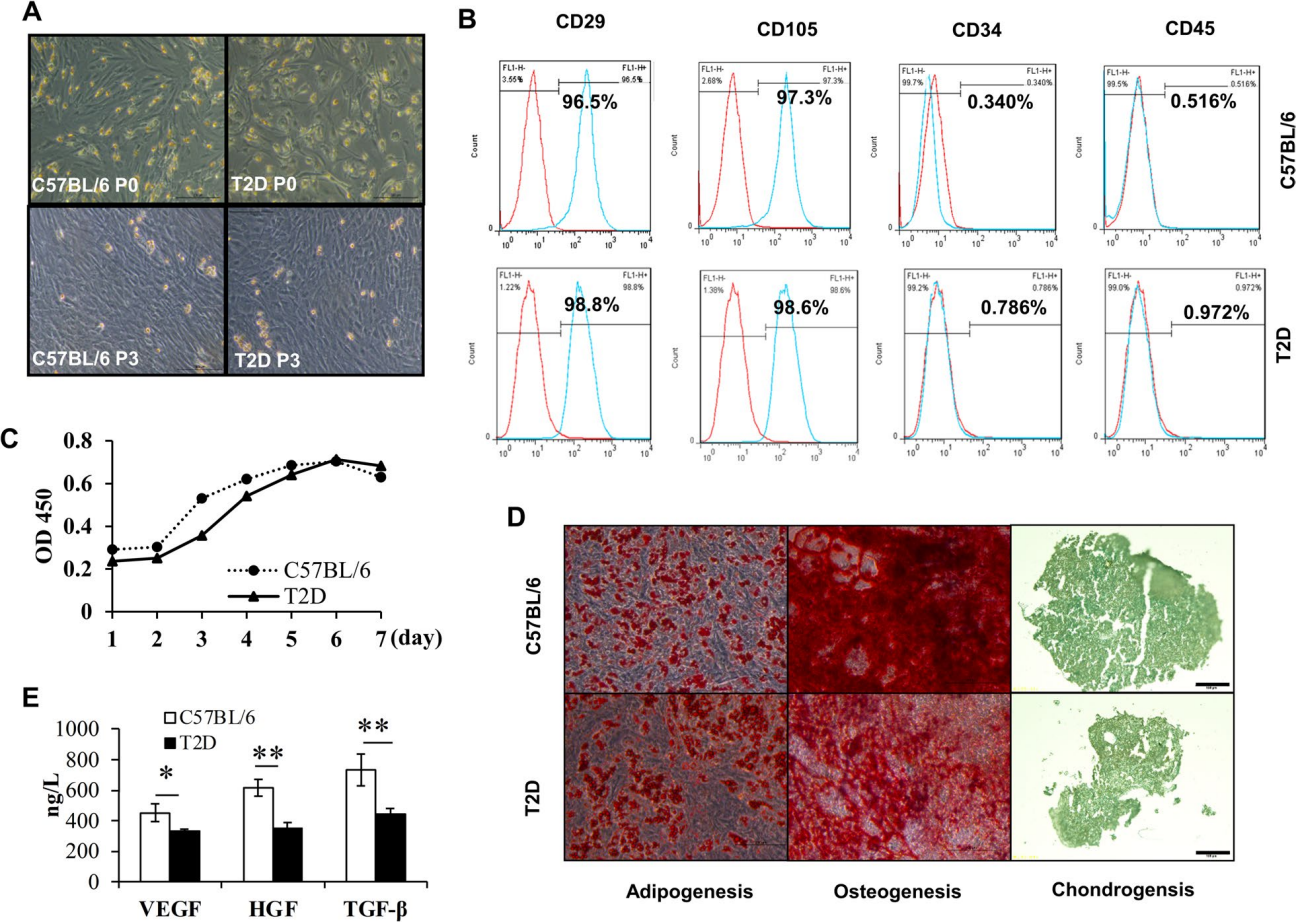
Characterization of ASCs isolated from C57BL/6 and T2D mice. **a** Representative micrographs of C57BL/6 and T2D ASCs at passage 0 and passage 3 observed under a light microscope. **b** Expression of CD29, CD105, CD34, and CD45 in ASCs harvested from C57BL/6L or T2D mice analyzed by flow cytometry. **c** Growth curves of C57BL/6 and T2D ASCs at passage 3. **d** The morphology of adipocytes, osteocytes, and chondrocytes derived from C57BL/6 ASCs and T2D ASCs identified by Oil Red, Alizarin Red, and Alcian Blue staining, respectively. Scale bar = 100 μm. **e** Concentrations of VEGF, HGF, and TGF-β secreted by C57BL/6 ASCs or T2D ASCs. Data are mean ± SEM of at least three individual experiments. At least 3 mice were included in each group. *P < 0.05, **P < 0.01, ANOVA test

